# Autistic adults exhibit a typical search advantage for facing dyads

**DOI:** 10.1002/aur.3265

**Published:** 2024-11-15

**Authors:** Tim Vestner, Bayparvah Kaur Gehdu, Katie L. H. Gray, Richard Cook

**Affiliations:** ^1^ School of Psychology Leeds Trinity University Leeds UK; ^2^ Department of Psychological Sciences, Birkbeck University of London London UK; ^3^ School of Psychology and Clinical Language Sciences University of Reading Reading UK; ^4^ School of Psychology University of Leeds Leeds UK

**Keywords:** autism, search advantage for facing dyads, social interactions, visual search

## Abstract

Recent findings obtained with non‐autistic participants indicate that pairs of facing individuals (face‐to‐face dyadic targets) are found faster than pairs of non‐facing individuals (back‐to‐back dyadic targets) when hidden among distractor pairings (e.g., pairs of individuals arranged face‐to‐back) in visual search displays. These results suggest that facing dyads may compete for observers' attention more effectively than non‐facing dyads. In principle, such an advantage might aid the detection of social interactions and facilitate social learning. Autistic individuals are known to exhibit differences in visual processing that impede their perception of other individuals. At present, however, little is known about multi‐actor visual processing in autism. Here, we sought to determine whether autistic individuals show a typical search advantage for facing dyads. In an online study, autistic and non‐autistic participants were tasked with finding target dyads (pairs of faces arranged face‐to‐face or back‐to‐back) embedded among distractor dyads (pairs of faces arranged face‐to‐back). Relative to the non‐autistic controls, the autistic participants took slightly longer to locate target dyads. However, a clear and comparable search advantage for facing dyads was seen in both participant groups. This preliminary evidence suggests that multi‐actor processing of autistic participants exhibits typical sensitivity to dyadic arrangement.

## Lay Summary

Recent findings obtained with non‐autistic participants indicate that pairs of people arranged face‐to‐face capture the attention of observers more effectively than pairs of people arranged back‐to‐back. The ability of these facing dyads to capture attention may help people locate social interactions in complex visual scenes. Here, we show that autistic participants also find facing pairs more noticeable than non‐facing pairs.

## INTRODUCTION

The visual perception of *individuals* has been an active area of study for many years. This line of research has identified dedicated neurocognitive mechanisms for the visual processing of faces (Duchaine & Yovel, 2015; Haxby et al., 2000), bodies (Peelen & Downing, 2007), and actions (Blake & Shiffrar, 2007). More recently, there has been growing interest in the perceptual mechanisms that allow us to interpret more complex social scenes depicting the interactions between people. In particular, pairs of individuals shown in arrangements that imply interaction, appear to engage neurocognitive processing that is not recruited by dyadic arrangements that imply non‐interaction (McMahon & Isik, 2023; Papeo, 2020; Quadflieg & Koldewyn, 2017).

An important behavioral finding in this emerging literature is the search advantage for facing dyads (Papeo et al., 2019; Vestner et al., 2019). When hidden among distractor dyads in visual search displays, participants locate face‐to‐face dyadic targets faster than back‐to‐back targets (Papeo et al., 2019; Vestner et al., 2019). Face‐to‐face targets are found faster than back‐to‐back targets when hidden among pairs of individuals that face in the same direction; that is, arranged face‐to‐back (Vestner et al., 2019). Similarly, face‐to‐face targets hidden among back‐to‐back distractors are found faster than back‐to‐back targets hidden among face‐to‐face distractors (Papeo et al., 2019).

According to one account, pairs of individuals arranged face‐to‐face capture observers' exogenous attention, while dyads arranged back‐to‐back do not (Papeo, 2020; Papeo & Abassi, 2019; Papeo et al., 2019). For example, it has been suggested that “facing dyads fall in the same biologically relevant category as faces or bodies, which are stimuli associated with high visual sensitivity, rapid discrimination, and spontaneous recruitment of attention” (Papeo et al., 2019, p. 1493). In principle, such an advantage might aid the detection of social interactions and facilitate social learning.

Many autistic individuals are known to exhibit differences in visual processing (Behrmann et al., 2006; Dakin & Frith, 2005; Simmons et al., 2009). These processing differences often impede autistic individuals' perception of others. In particular, many autistic people experience difficulties when asked to identify faces (Gehdu et al., 2022; Hedley et al., 2011; Stantić et al., 2021). These difficulties seem to extend to some other facets of social vision including the perception of facial motion (Keating et al., 2022; Shah et al., 2016), the discrimination of static body postures (Reed et al., 2007), and the interpretation of whole‐body actions (Atkinson, 2009).

At present, however, little is known about multi‐actor visual processing in autism. Here, we sought to determine whether autistic individuals show a typical search advantage for facing dyads. In light of other social vision difficulties described previously (e.g., Atkinson, 2009; Gehdu et al., 2022; Hedley et al., 2011; Keating et al., 2022; Reed et al., 2007; Shah et al., 2016; Stantić et al., 2021), we reasoned that autistic observers might exhibit aberrant multi‐actor processing, and thus fail to demonstrate a search advantage for facing dyads.

In an online study, autistic and non‐autistic participants were tasked with finding target dyads (pairs of faces arranged face‐to‐face or back‐to‐back) embedded among leftward‐ and rightward‐facing distractor pairs arranged face‐to‐back. In this paradigm, the search advantage for facing dyads is evidenced by shorter response times (RTs) when searching for face‐to‐face targets than when searching for back‐to‐back targets (Flavell et al., 2022; Vestner et al., 2020; Vestne et al., 2021; Vestner et al., 2021; Vestner et al., 2019).

## METHODS

### Participants

Forty‐eight participants with a clinical diagnosis of autism (*M*_age_ = 36.6 years; *SD*_*a*ge_ = 11.9 years) were recruited via www.ukautismresearch.org. All autistic participants had received an autism diagnosis (e.g., Autism Spectrum Disorder, Asperger's Syndrome) from a clinical professional (General Practitioner, Neurologist, Psychiatrist or Clinical Psychologist) based in the United Kingdom. Of the 48 autistic participants, 10 described their sex as male and 38 described their sex as female. Forty‐seven identified as White (44: White‐British, 1: White‐Irish, 2: White‐Other). The remaining autistic participant did not specify their ethnicity. All participants in the autistic group reached cut‐off (a score of 32 or more) on the Autism‐Spectrum Quotient (AQ; *M* = 42.25, *SD* = 4.10; Baron‐Cohen et al., 2001).

Forty‐eight non‐autistic individuals (*M*_age_ = 38.1 years; *SD*_*a*ge_ = 11.2 years) were recruited via www.prolific.com to serve as controls. Of the 48 non‐autistic controls, 11 described their sex as male and 37 described their sex as female. Forty‐four identified as White (43: White‐British, 1: White‐Irish), 2 identified as Black‐British, and 2 as British‐Asian. All non‐autistic participants scored below cut‐off (a score of 31 or less) on the AQ (*M* = 21.85, *SD* = 4.46).

The autistic and non‐autistic participants did not differ significantly in terms of age (*t*[94] = 0.637, *p* = 0.526) or sex (*X* ^2^_[1]_ = 0.061, *p* = 0.805). To be eligible, all participants had to be aged between 18 and 60, speak English as a first language, and have normal or corrected‐to‐normal visual acuity. All participants were required to be a current UK resident.

Data collection for the study took place between June and August 2023. At the outset, our aim was (i) to recruit as many autistic participants as possible during this period, and (ii) to stop data collection at the end of August provided a minimum sample size of *N* = 44 had been reached. Two groups of *N* = 44 yield an 85.4% chance of detecting a moderate‐to‐large effect (*d* = 0.65) when using an independent‐samples *t*‐test (α = 0.05, two‐tailed) to compare ΔRT_autistic_ and ΔRT_non‐autistic_. Our final sample (*N* = 48) exceeded this minimum. With two groups of *N* = 48, post‐hoc power analysis indicated an 88.3% chance of detecting a moderate‐to‐large effect size (*d* = 0.65).

To ensure that the autistic and non‐autistic participants were approximately matched for non‐verbal intelligence, all participants completed a matrix reasoning task (MRT). Forty items were selected from the Matrix Reasoning Item Bank (MaRs‐IB; Chierchia et al., 2019). Participants were given 30 seconds to complete each puzzle by selecting the correct answer from 4 options. The scores of the autistic (*M* = 25.15, *SD* = 6.20, range: 13–38) and non‐autistic participants (*M* = 26.42, *SD* = 5.74, range: 14–36) did not differ significantly [*t*(94) = 0.947, *p* = 0.346].

The presence of alexithymia was assessed in all participants using the 20‐item Toronto Alexithymia Scale (TAS‐20; Bagby et al., 1994; Taylor et al., 2003). We sought to measure alexithymia – a trait associated with diminished ability to interpret affective and interoceptive states (Bird & Cook, 2013)–in light of evidence that this trait affects how individuals inspect faces (Cuve et al., 2021). As expected, the TAS‐20 scores of the autistic participants (*M* = 66.27, *SD* = 13.53, range: 35–94) were significantly higher than those of the non‐autistic controls (*M* = 44.98, *SD* = 13.11, range: 24–76) (*t*[94] = 7.828, *p* < 0.001).

We also assessed the face recognition of all participants using the Australian variant of Cambridge Face Memory Test (CFMT‐A; McKone et al., 2011) and the 20‐Item Prosopagnosia Index (PI20; Shah et al., 2015; Tsantani et al., 2021). The CFMT‐A scores of the autistic participants (*M* = 71.47%, *SD* = 13.75%, range: 43.1%–95.8%) were significantly lower than those of the non‐autistic controls (*M* = 79.03%, *SD* = 9.74, range: 58.3%–98.6%) (*t*[94] = 3.107, *p =* 0.003). Similarly, the PI20 scores of the autistic participants (*M* = 61.54, *SD* = 20.95, range: 24–98) were significantly greater than those of the non‐autistic controls (*M* = 46.25, *SD* = 13.85, range: 22–84) (*t*[94] = 4.218, *p <* 0.001). Both results indicate superior face recognition in the non‐autistic group relative to the autistic group.

### Dyad search task

**FIGURE 1:**
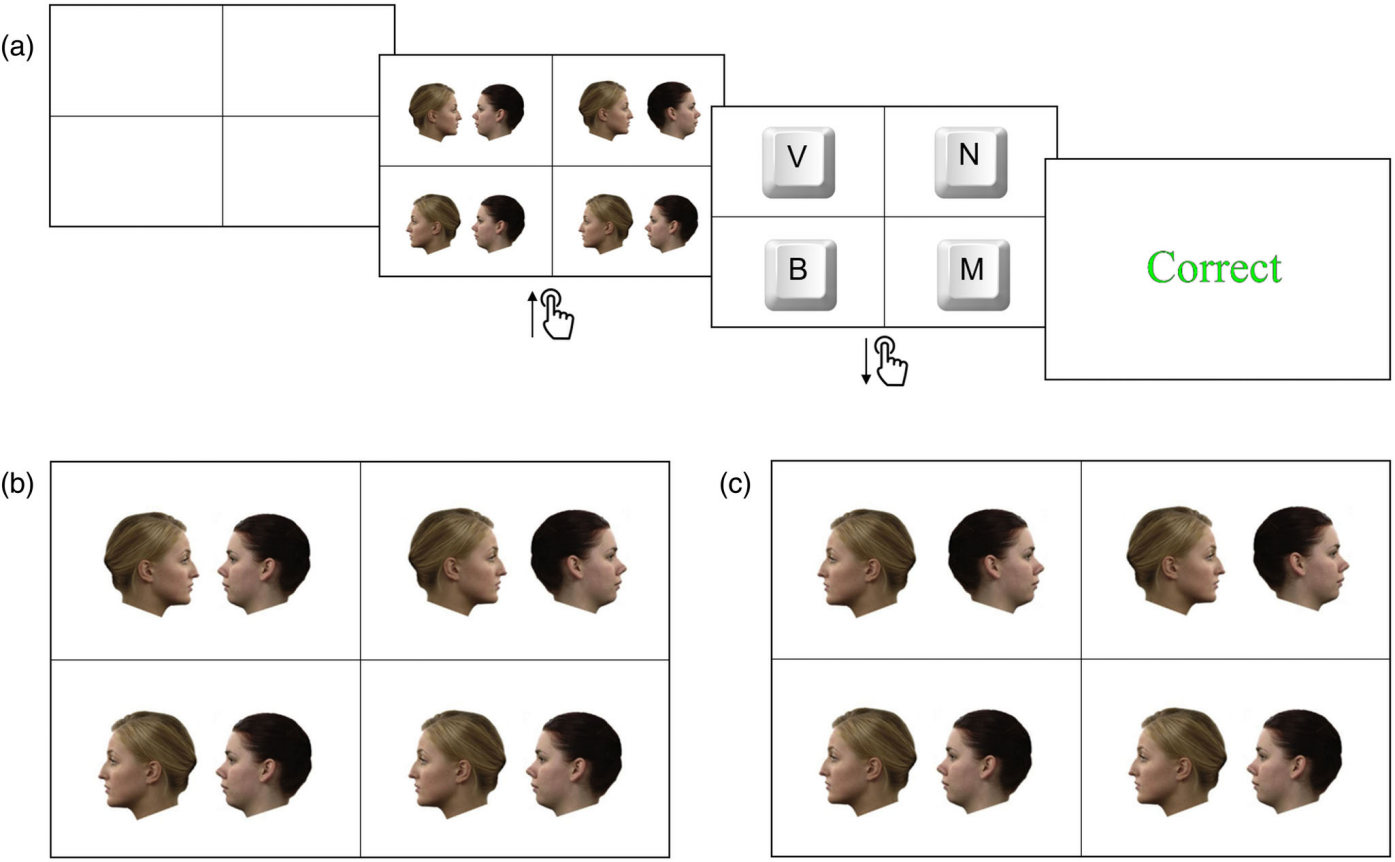
Overview of the dyadic search task. (a) Schematic illustration of a trial sequence. (b) Illustration of a search display from the facing condition. (c) Illustration of a search display from the non‐facing condition.

Each trial started with a black cross that divided the white display into four quadrants (Figure 1a). Participants initiated the onset of the search array by pressing and holding down spacebar. While spacebar was held down, pairs of individuals appeared in the four quadrants. Each dyad was made up of two same‐sex faces viewed in profile, chosen randomly from the pool of stimuli by the experimental program. The same two individuals featured in all four dyads presented on a given trial.

One of the four dyads was the target. In one block, target dyads were arranged face‐to‐face (Figure 1b); in the other, target dyads were arranged back‐to‐back (Figure 1c). The remaining three dyads were distractors. Both individuals depicted in the distractor dyads were always shown looking in the same direction, either leftwards or rightwards (i.e., arranged face‐to‐back). Each trial featured at least one distractor pair facing left and one facing right. Within each group, 24 participants completed the face‐to‐face block first, while 24 completed the back‐to‐back block first.

Participants were instructed to let go of spacebar as soon as they found the target dyad. As soon as they let go, the search array was replaced by a display prompting participants to identify the target location by making one of four keypress responses. Participants were therefore unable to continue their search after the release of the spacebar (Meegan & Tipper, 1998). RTs measure the interval from when spacebar was pressed to when it was released. If no response was recorded within 5 seconds, the search display disappeared, and an incorrect response registered.

Each block consisted of 50 trials: 45 experimental trials plus 5 catch trials. On catch trials no target dyad was presented. Instead, search arrays comprised two leftwards facing dyads, and two rightwards facing dyads. On catch trials, participants were instructed to hold down spacebar until all the pairs disappeared (after 5 seconds). Catch trials were included to discourage participants from releasing spacebar before the target pair had been found on test trials. Participants who failed to respond correctly on at least five of the 10 catch trials were replaced (one non‐autistic control was replaced on this basis).

The eight facial images used (four female, four male) were sourced from the Radboud Face Database (Langner et al., 2010). All images were normed to a height of 350 pixels. For each image, a mirror‐image was created so that a given individual could appear facing left or right.

Participants completed the dyad search task online. The experiment was coded using Unity3D (Version 2018.3.7f1), compiled to WebGL and hosted on an Amazon Lightsail server. This allowed the experiment to run in a participant's browser and RTs to be recorded locally without being influenced by variations in data transmission speed to the server. We have previously confirmed that this online procedure produces similar RT distributions to those seen in the lab (Vestner et al., 2020). The data supporting all analyses can be accessed via OSF (https://osf.io/cegx9/).

## RESULTS

Overall task performance was good. The non‐autistic controls responded correctly on 95.8% of experimental trials (range: 83.3%–100.0%) and 92.5% of catch trials (range: 50%–100%). The autistic participants responded correctly on 95.0% of experimental trials (range: 85.6%–100.0%) and 91.5% of catch trials (range: 60%–100%). When calculating each participant's mean RT in the face‐to‐face and back‐to‐back conditions, we excluded trials where they responded incorrectly. No other data points were removed. The proportion of correct responses on experimental trials (i.e., the number of valid datapoints) did not differ significantly between the groups (*t*[94] = 0.154, *p* = 0.390). Similarly, there was no group difference in terms of catch‐trial performance (*t*[94] = 0.497, *p* = 0.620).

**FIGURE 2:**
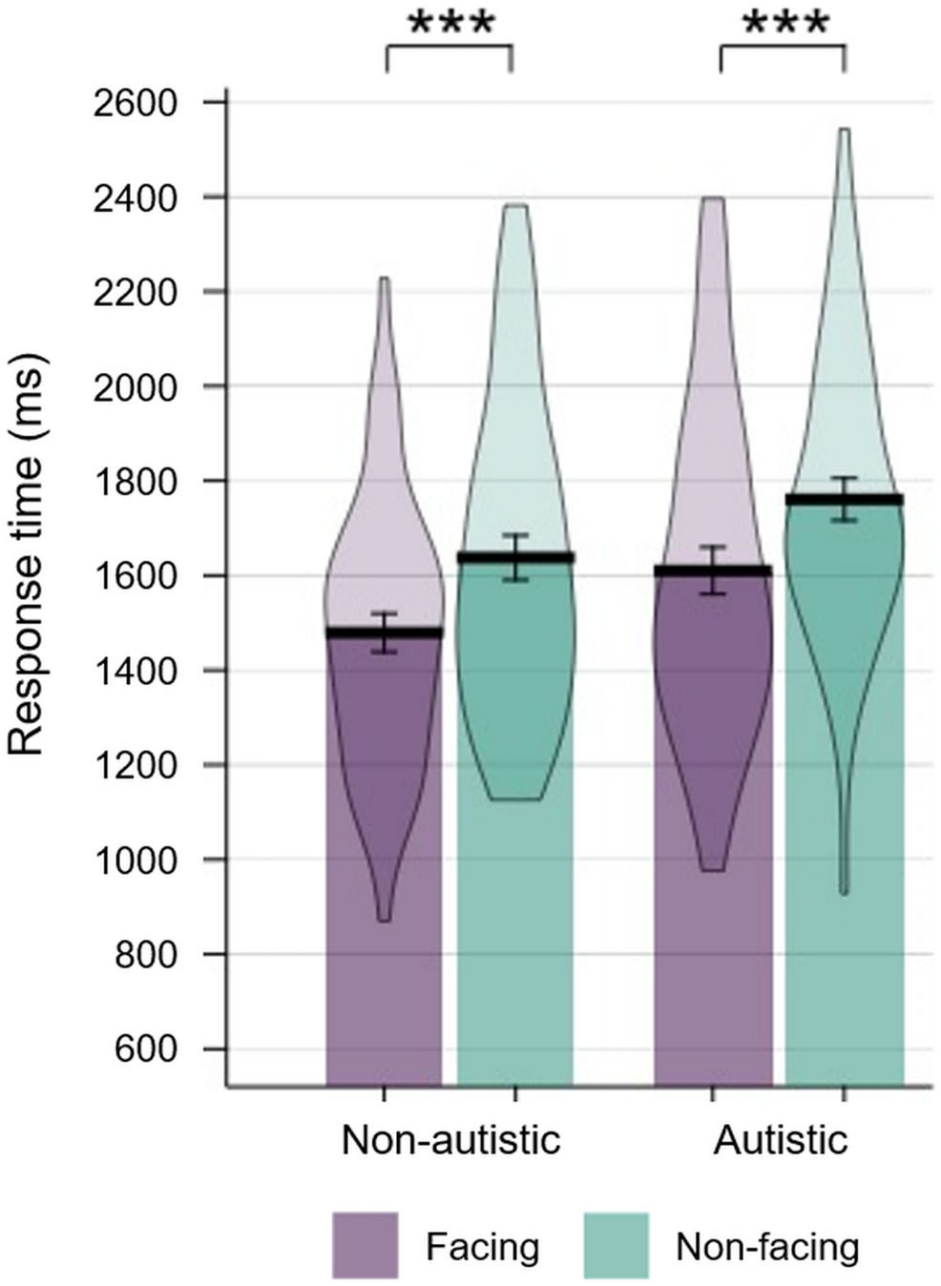
Results of the dyadic search experiment. Error bars denote ± SEM. ***denotes *p* < 0.001.

Participants' RTs on the dyad search task were subjected to ANOVA with Target Type (face‐to‐face, back‐to‐back) as a within‐subjects factor and Group (autistic, non‐autistic) as a between‐subjects factor (Figure 2). The analysis revealed a significant main effect of Group (*F*[1,94] = 4.562, *p* = 0.035, η_p_^2^ = 0.046), whereby the autistic participants responded slower than the non‐autistic participants. We also observed a main effect of Target Type (*F*[1,94] = 38.760, *p* < 0.001, η_p_^2^ = 0.292), whereby participants responded faster in the face‐to‐face target condition than in the back‐to‐back target condition.

Crucially, the analysis revealed no Group × Target Type interaction (*F*[1,94] = 0.024, *p* = 0.878, η_p_^2^ < 0.001). A highly significant search advantage for facing dyads was seen in both the autistic group (*M* = 151 ms, *SD* = 259 ms; *t*[47] = 4.023, *p* < 0.001, *d* = 0.581) and non‐autistic group (*M* = 158 ms, *SD* = 226 ms; *t*[47] = 4.861, *p* < 0.001, *d* = 0.702). Bayesian analysis of these distributions conducted in JASP (JASP‐Team, 2022) yielded substantial evidence (BF_01_ > 3.0; Jeffreys, 1961) for the null hypothesis (*t*[94] = 0.154, *p* = 0.878, BF_01_ = 4.61); that is, that the search advantage for facing dyads does not differ as a function of Group. This Bayesian *t*‐test was conducted using the default Cauchy priors (center = 0, width = 0.707).

No correlations were observed, either in the autistic or non‐autistic group, between individuals' susceptibility to the search advantage for facing dyads and their scores on the AQ, the TAS‐20, the MRT, the CFMT‐A or the PI20 (Table 1). In the autistic group, there was a significant positive correlation between performance on the MRT and the proportion of correct responses made on the search task (*r*_p_ = 0.619, *p* < 0.001), that survived Bonferroni correction for multiple comparisons. In the non‐autistic group, there was a weak correlation between individuals' PI20 scores and proportion of correct responses on the search task (*r*_p_ = 0.287, *p* = 0.048), but this did not survive correction for multiple comparisons. No other significant correlations were observed.

**TABLE 1:** Correlations (*r*_p_) observed between (i) individuals' susceptibility to the search advantage for facing dyads (ΔRT) and (ii) their accuracy on the search task (% Correct), and their scores on the autism‐spectrum quotient (AQ), the 20‐item Toronto Alexithymia Scale (TAS‐20), the matrix reasoning task (MRT), the Australian variant of the Cambridge face memory test (CFMT‐A) and the 20‐Item prosopagnosia index (PI20). *** denotes *p* < 0.001. *denotes *p* < 0.05.

	Autistic (*N* = 48)		Non‐autistic (*N* = 48)	
	ΔRT	% Correct	ΔRT	% Correct
AQ	−0.163	0.118	0.052	−0.203
TAS‐20	−0.046	0.156	−0.052	0.259
MRT	0.114	0.619***	−0.243	0.248
CFMT‐A	0.000	0.157	−0.149	−0.001
PI20	0.072	0.171	0.125	0.287*

## GENERAL DISCUSSION

In the present study, we sought to determine whether autistic individuals exhibit a search advantage for facing dyads – a key behavioral effect in the emerging literature on multi‐actor visual processing (Papeo et al., 2019; Vestner et al., 2019). Both our autistic and non‐autistic participants exhibited this effect: face‐to‐face targets were found faster than back‐to‐back targets when embedded among leftward‐ and rightward‐facing distractor pairs arranged face‐to‐back. There was little or no sign that the magnitude of the search advantage differed in the two groups.

At present, relatively little is known about multi‐actor visual processing in autism. When non‐autistic observers view pairs of individuals in profile, it has been suggested that face‐to‐face arrangements engage domain‐specific visual processing—thought to afford high levels of visual sensitivity, rapid discrimination, and the spontaneous recruitment of attention—that is not recruited by back‐to‐back arrangements (Papeo, 2020; Papeo & Abassi, 2019; Papeo et al., 2017; Papeo et al., 2019). The preliminary evidence described here suggests that multi‐actor processing of autistic participants may exhibit typical modulation by arrangement, potentially consistent with the differential processing of face‐to‐face and back‐to‐back dyads.

Broadly speaking, the results described here accord with recent evidence that autistic and non‐autistic participants exhibit comparable sensitivity to changes in inter‐actor distance when viewing face‐to‐face dyadic arrangements from third‐person perspectives (Bunce et al., 2024). If putative domain‐specific visual processing—selectively recruited by facing dyads—were aberrant in autistic participants, one would expect to see diminished perceptual sensitivity to configural attributes such as inter‐actor distance. However, the findings of Bunce et al. (2024) argue against this possibility. Our results are also consistent with recent evidence that neural markers (obtained via EEG) thought to index the recognition of social interactions (Oomen et al., 2022), do not differ in autistic and non‐autistic observers (Oomen et al., 2023).

Compared to the non‐autistic controls, our autistic participants took slightly longer to identify the location of target dyads than our non‐autistic participants. Note, however, this main effect of Group did not interact with Target Type (face‐to‐face vs. back‐to‐back). As described above, our autistic and non‐autistic participants showed a clear and comparable search advantage for facing dyads. One possibility is that multi‐actor processing—of both facing and non‐facing arrangements—is less efficient in autistic participants. A second possibility is that apparent inefficiency in the visual processing of dyadic arrangements may be a knock‐on consequence of difficulties encoding the constituent actors. Perceptual representations of individual actors are the ‘building blocks’ that underpin higher‐level representations of dyadic interactions (akin to the relationship between letter and word representations). This account accords well with the fact that our autistic participants exhibited less accurate face recognition than our non‐autistic participants.

In the present study, we used profile views of faces/heads to create the face‐to‐face, face‐to‐back, and back‐to‐back dyadic stimuli employed in our search displays. Previous research has repeatedly demonstrated that this kind of stimulus produces a clear and robust search advantage for facing dyads (Vestner et al., 2020; Vestner et al., 2021; Vestner et al., 2021). We acknowledge, however, that other kinds of stimuli also produce the search advantage, notably dyads constructed from images of whole bodies (viewed in profile) in neutral (e.g., Vestner et al., 2019) and active poses (e.g., Papeo et al., 2019). To date, there is no evidence that the search advantage for facing dyads elicited by arrangements constructed from heads/faces differs qualitatively from that elicited by whole‐body stimuli. Nevertheless, there may be some value in replicating the present results using whole‐body stimuli.

In a similar vein, it might also be interesting to examine the effects of a set‐size manipulation of the performance of autistic and non‐autistic participants. The quadrant search task employed here (i.e., one target dyad hidden among three distractor dyads) produces a clear and robust search advantage for facing dyads with both whole‐body and head/face stimuli (Papeo et al., 2019; Vestner et al., 2019; Vestner et al., 2020; Vestner et al., 2021; Vestner et al., 2021). However, it is possible that increasing the set‐size (i.e., the number of distractor dyads present in the search display) may yet reveal subtle differences between autistic and non‐autistic observers.

Consistent with several recent studies that have sought to recruit autistic participants online, our sample included a large proportion of female participants (Rødgaard et al., 2022). Consequently, we acknowledge the need to replicate the preliminary findings described here in a sample more representative of the wider autistic population, the majority of whom identify as male (e.g., Lai et al., 2015).

## CONCLUSION

To date, relatively little is known about multi‐actor visual processing in autism. Here, using a Bayesian analysis, we show that autistic and non‐autistic observers exhibit a comparable search advantage for facing dyads. This preliminary evidence suggests that multi‐actor processing of autistic participants exhibits typical sensitivity to face‐to‐face vs. back‐to‐back dyadic arrangement.

## ETHICS STATEMENT

Ethical clearance was granted by the Departmental Ethics Committee for Psychological Sciences, Birkbeck, University of London. The experiment was conducted in line with the ethical guidelines laid down in the 6th (2008) Declaration of Helsinki. All participants gave informed consent.

## Data Availability

The data that support the findings of this study are openly available in OSF at https://osf.io/cegx9/.
